# A nasal spray vaccination device based on Laval nozzle and its experimental test

**DOI:** 10.1038/s41598-023-33452-0

**Published:** 2023-04-17

**Authors:** Zhong Wang, Zhengyuan Zhang, Qian Wang, Lingliao Zeng, Jian Jin

**Affiliations:** 1 Guangzhou Laboratory, Guangzhou, 510005 China; 2 Guangzhou Institutes of Biomedicine and Health, Chinese Academy of Sciences, Guangzhou, 510530 China; 3 State Key Laboratory of Respiratory Disease, Guangzhou Institute of Respiratory Health, the First Affiliated Hospital of Guangzhou Medical University, Guangzhou, 510182 China; 4 School of Mechanical Engineering, Hefei University of Technology, Hefei, 230009 China

**Keywords:** Vaccines, Biomedical engineering, Mechanical engineering

## Abstract

In order to realize the application of the nasal spray vaccination in the prevention and protection of respiratory infectious diseases, a nasal spray vaccination device is designed in this paper. The device uses a Laval nozzle structure to generate a high-speed airflow that impinges on the vaccine reagent and forms nebulized particles. Through optimizing of the Laval nozzle structure and testing experiments on spray particle size, spray velocity, spray angle and spray rate, a set of parameters which is applicable to actual nasal spray vaccination is obtained. The experimental results show that when the air source pressure is 2 bar, the spray angle is about 15°, the diameter of the spray particles Dv50 is about 17 μm, the volume fraction of particles with diameter smaller than 10um is about 24%, the spray rate is close to 300 μl/s. The vaccine activity tests demonstrate that under these conditions, not only the biological activity of vaccines is guaranteed, but also the delivery efficiency is well assured.

## Introduction

Compared with traditional intramuscular vaccination, nasal spray vaccination has more advantages in terms of cost and ease of administration^1^, and is easier to be accepted as noninvasive. Relevant studies have shown that nasal spray vaccination can provide effective protection against respiratory infectious diseases^2–4^, and are superior to intramuscular injections in terms of effectiveness of immune protection^5–8^.

The nebulized drug delivery method has been widely used in hospitals^9,10^. According to the principle of nebulization, they can be mainly divided into three types: ultrasonic nebulization, vibrating-mesh nebulization and jet nebulization^11^. Unlike the general nebulization drug delivery process, the administration of nasal spray vaccination requires the use of biologically active vaccine reagents; therefore, nebulization methods that affect vaccine activity, such as ultrasonic nebulization is not suitable for nasal spray vaccination^9–12^. Vibrating-mesh nebulizer produce particles with a size of 3–5 μm^9^, and this is suitable for nebulization of drugs that require lung deposition^13^. The maximum nebulization rate of common jet nebulizer is generally less than 10 μl/s^14^.

From a pharmaceutical viewpoint, the size distribution of the sprayed particles/droplets is one of the most important parameters for an inhalation product^15^. Since particle size in the range of 2–10 μm can cause lung deposition^16^, for nasal sprays, the size of most spray particles should be greater than 10 μm^17^, and for particles with diameter range from 5 to 50 μm, the particles of about 10 μm are most easily to be absorbed by the nasal cavity^18^. At the same time, there are certain requirements for the amount of vaccine to ensure the success of immunization. For example, the Ad5-S vaccine used in this paper has a vaccine dosage requirement of 400 μl per dose. For mass vaccination situations such as COVID-19 vaccination, a high nebulization rate is helpful to save time for each vaccination.

Therefore, this required a spray device that produced particles larger than 10 μm in size, the spray particles Dv50 is close to 10 μm, and the spray rate could be relatively high. Thus, we developed a Laval nozzle based nasal spray vaccination device. After optimization of the Laval nozzle structure and experimental parameters, a good nebulization result was obtained and the vaccine activity tests also proved the effectiveness of this device.

## Method

### System design

**Figure 1:**
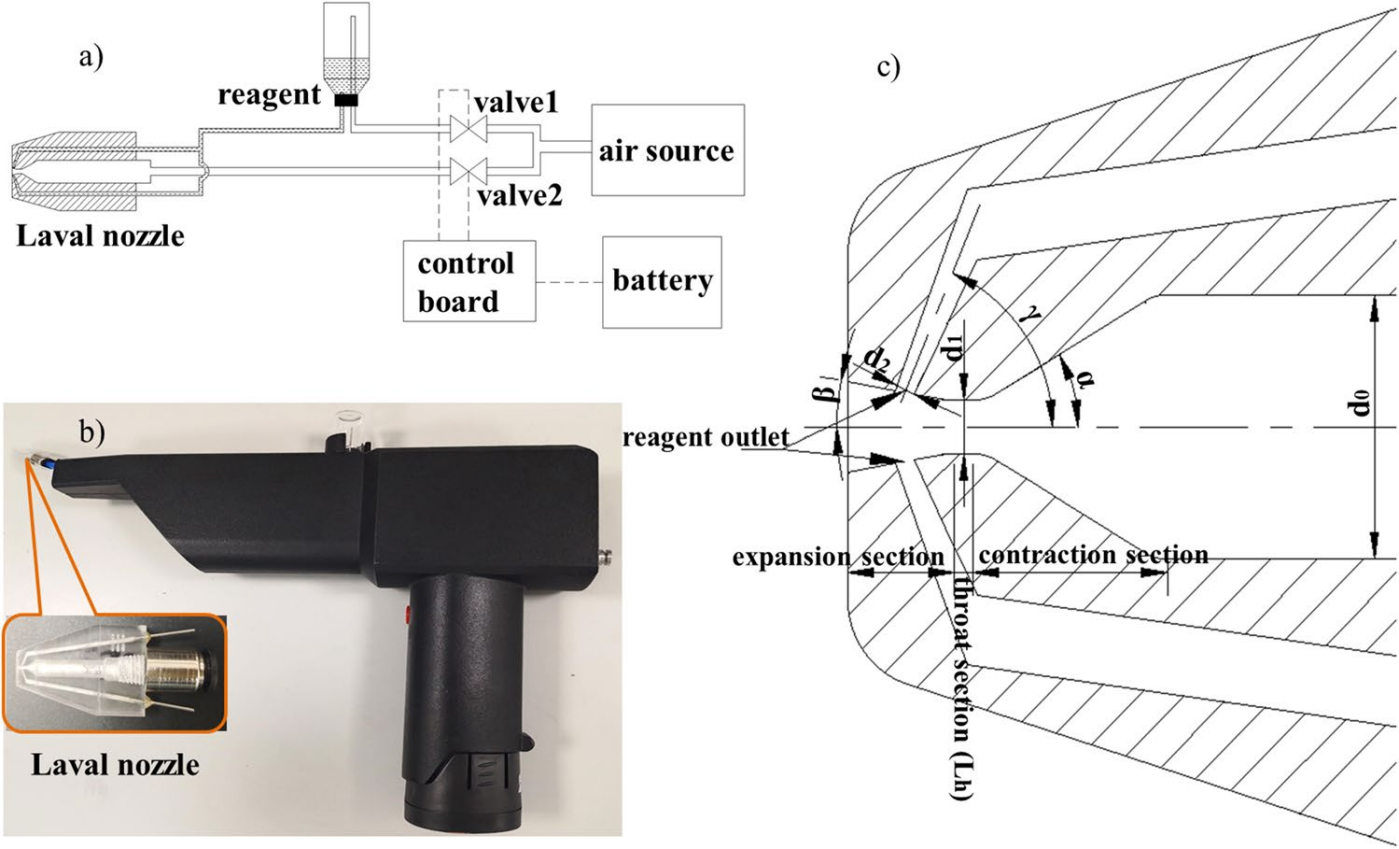
(**a**) Schematic diagram of the system; (**b**) the prototype of the device; (**c**) structure of the Laval nozzle.

Figure 1a is the schematic diagram of the system. The pressure of the two channels is controlled by two solenoid valves. One channel of pressure air propels the vaccine reagent to the Laval nozzle, the other one directly reaches to the Laval nozzle. The vaccine reagent and the pressure air met at the Laval nozzle and will be ejected from the nozzle in the form of nebulized particles. Aim the nozzle of the device at the nostrils of the vaccinee and press the spray button, the nasal spray vaccination process is completed.

Figure 1b is the prototype of the device. An external nitrogen cylinder is used as air source, the solenoid valves are two-position three-way normal closed solenoid valve, and the Laval nozzle was fabricated by 3D printer ASIGA-MAX X27UV (Asiga, Alexandria, New South Wales, Australia).

The delivery efficiency of nasal spray vaccine is directly affected by factors such as spray particle size, spray rate and spray angle. By adjusting the internal structure of Laval nozzle and controlling the air source pressure, the above parameters can be precisely regulated. By integrating with external air source and solenoid valve and supplemented with the control system, the device can be convenient and easy to use by medical staff.

### Design of the Laval nozzle

In this device, the size of the spray particle is mainly determined by the pressure of the air source and the structure of the nozzle. The nebulization principle is as follows: two liquid reagents collide at a certain angle at the intersection of channels, and meanwhile, the high-speed airflow impinges on the liquid reagent to form nebulized particles and eject out from the nozzle.

The high-speed airflow has a crucial influence on the size of the spray particles. In order to generate high-speed airflow, the nozzle outlet is designed to be a Laval structure, as shown in Fig. 1c. The Laval structure consists of three parts: contraction section, throat section and expansion section^19,20^. Driven by pressure, the low-speed airflow from the contraction section reaches the throat section. The airflow velocity will be greatly increased due to the significant reduction of the throat diameter^21–23^.

### Influence on spray particle size

The diameter of the central tube d_0_ is a constant value, which is determined by the size of the air connector. Here, d_0_ is set to 3 mm. Structure parameters that affect the nebulization result of the Laval nozzle mainly includes: semi-cone angle of contraction section (α), throat diameter (d_1_), throat length (L_h_), reagent outlet diameter (d_2_), reagent outlet angle (γ), semi-cone angle of expansion section (β). In order to analyze the influence of the six parameters on the nebulization result, the orthogonal experimental method is adopted. Five levels for each parameter and ${\text{L}}_{25} \left( {5^{6} } \right)$ orthogonal table are used for the experimental test.

According to experience, the semi-cone angle of contraction section α is generally around 30°^24^. The semi-cone angle of expansion section β is in the range of 8°–15°^25^. Theoretically, the larger the ratio of central pipe diameter d_0_ to throat diameter d_1_ is, the better to nebulization^26^. As long as the cross sections of the contraction section and expansion section are smooth, the throat length L_h_ can be 0^24^. Combined with the actual situation of nasal spray vaccination, the 5 levels selected for each parameter are shown in Table 1.

**Table 1:** Structural factors and levels of Laval nozzle.

Level	Factors					
	α/°	d_1_/mm	L_h_/mm	d_2_/mm	γ/°	β/°
1	20	0.4	0.1	0.1	55	8
2	25	0.5	0.2	0.15	60	10
3	30	0.6	0.3	0.2	65	12
4	35	0.7	0.4	0.25	70	14
5	40	0.8	0.5	0.3	75	16

**Figure 2:**
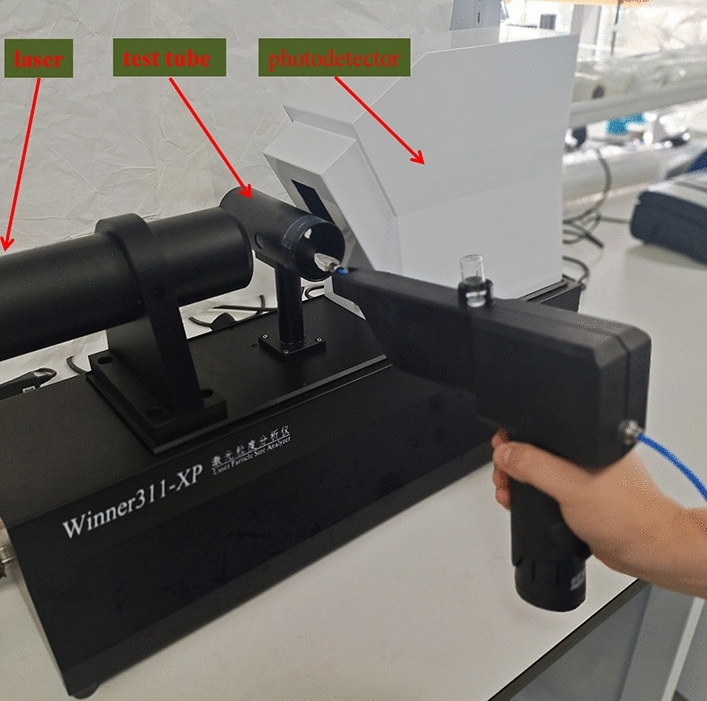
The experimental diagram.

The orthogonal experiments are performed using the diameter of the spray particles as the test index. Winner311-XP system (Jinan Micro and Nano Particle Instrument Co., Ltd. Jinan, China) is used to measure the nebulization diameter of the particles. The instrument control, experiment setup, and data analysis are all performed by the Jinan Winner Software (Version 3.0, Jinan Micro and Nano Particle Instrument Co., Ltd. Jinan, China). The Winner311-XP is a dynamic automated imaging system combined with Raman spectroscopy, a laser with a wavelength of 650 nm is used in it, it’s capable to measure particles with a diameter of 0.1–100 μm. During the test, the nozzle outlet is kept at the center of the end face of the analyzer’s test tube, it is about 6 cm away from the measure area. Experimental diagram is shown in Fig. 2. Water is used as the nebulization reagent and the outlet pressure of the nitrogen source is set to 2 bar.

### Influence on spray velocity

For nasal spray vaccination, the high spray rate tends to cause nasal discomfort in vaccinees. Therefore, it is better to reduce the spray velocity while ensuring the efficiency of vaccination.

The κ-ε model of COMSOL software is used to simulate the airflow in the Laval nozzle and the area within 15 mm off the Laval nozzle. Since the air velocity determines the spray velocity, only the airflow field distribution is simulated and the air velocity is used to reflect the spray velocity. We assume that the distance between the Laval nozzle and nostril is 2 mm. So a point 2 mm away from the nozzle outlet is chosen as the measure point to reflect the air velocity at the nostril.

### Influence on spray angle

To facilitate the nasal spray vaccine into nostril, the angle of the spray nozzle should be as small as possible. Among the related parameters of the Laval nozzle structure, the semi-cone angle of expansion section β is the main parameter that causes the change of spray angle. For studying the effect of different β on the spray angle, the spray angles of Laval nozzles were measured by means of photographs for β of 8°, 10°, 12°, 14° and 16°.

### Vaccine activity test

Ad5-S vaccine is used to test the affection of the device under 2 bar air source pressure. Infection-forming units (IFU) can be used to evaluate the activity of viral vectored vaccines, and the test is as follows. In brief, human embryonic kidney cells HEK293 were seeded into 24-well plates at 2.5 × 10^5^ cells per well. Serially tenfold diluted Ad5-S vaccine (provided by research group in Guangzhou Institutes of Biomedicine and Health, Chinese Academy of Health) was added to each well immediately and cultured for 48 h at 37 °C. Subsequently, the cells were fixed with anhydrous methanol, washed with PBS supplemented with 1% bovine serum albumin (BSA, BBI, A600332-0100), and incubated with anti-Ad5 rabbit serum (1:500 in PBS supplemented with 1% BSA) for 1 h at 37 °C. Then, the wells were washed and incubated with horseradish peroxidase (HRP)-conjugated goat anti-rabbit IgG antibodies (KPL, 5220-0336, 1:500 in PBS supplemented with 1% BSA). 1 h later, after incubated for 1 h at 37 °C, the wells were washed and developed with 1 × DAB solution (MCE, HY-15912/CS-3030) for 10 min at room temperature. Removed the DAB solution and added PBS to terminate the color development. Count the colored spots under microscope to calculate the IFU, which equals spots per field times fields per well divide by the product of vaccine volume times dilution gradient.

## Result

### Orthogonal experimental result

The orthogonal experimental results are shown in Table 2. The spray particle diameter when the volume fraction of the nebulized particles reaches 50% (Dv50) is taken as the recorded value, each test result is obtained from the average of 5 sets of experimental data.

**Table 2:** The orthogonal test results.

No.	A	B	C	D	E	F	Dv50/um
	α/°	d_1_/mm	L_h_/mm	d_2_/mm	γ/°	β/°	
1	1	1	1	1	1	1	25.36
2	1	2	2	2	2	2	23.15
3	1	3	3	3	3	3	25.89
4	1	4	4	4	4	4	24.55
5	1	5	5	5	5	5	26.76
6	2	1	2	3	4	5	17.75
7	2	2	3	4	5	1	21.05
8	2	3	4	5	1	2	23.49
9	2	4	5	1	2	3	22.60
10	2	5	1	2	3	4	29.56
11	3	1	3	5	2	4	24.24
12	3	2	4	1	3	5	14.27
13	3	3	5	2	4	1	17.73
14	3	4	1	3	5	2	31.61
15	3	5	2	4	1	3	30.91
16	4	1	4	2	5	3	13.81
17	4	2	5	3	1	4	16.75
18	4	3	1	4	2	5	18.83
19	4	4	2	5	3	1	31.41
20	4	5	3	1	4	2	29.93
21	5	1	5	4	3	2	26.50
22	5	2	1	5	4	3	24.67
23	5	3	2	1	5	4	16.61
24	5	4	3	2	1	5	24.72
25	5	5	4	3	2	1	35.53
K1	125.70	107.65	130.03	108.78	121.23	131.08	∑K = 597.68
K2	114.45	99.90	119.83	108.96	124.35	134.67	
K3	118.76	102.55	125.83	127.52	127.63	117.89	
K4	110.73	134.88	111.65	121.84	114.62	111.71	
K5	128.03	152.70	110.34	130.57	109.85	102.33	
R	17.30	52.79	19.68	21.79	17.78	32.34	–

As can be seen from Table 2, the max factors difference R of each factor from largest to smallest is B, F, D, C, E, and A. Therefore, d_1_ (throat diameter) has the greatest effect on the spray particle diameter Dv50, β (semi-cone angle of expansion section) is second, while d_2_ (reagent outlet diameter), γ (reagent outlet angle) and L_h_ (throat length), α (semi-cone angle of contraction section) has less influence. The results of the orthogonal experiment indicate that the optimal solution is A4B2C5D1E5F5, that is: α = 35°, d_1_ = 0.5 mm, L_h_ = 0.5 mm, d_2_ = 0.1 mm, γ = 75°, β = 16°.

### Spray velocity simulation

According to the results of the orthogonal experiments in Table 2, throat diameter d_1_ has a significant effect on the nebulized particle diameter Dv50. However, the results of K2 and K3 of throat diameter d_1_ (factor B) are 99.90 and 102.55, respectively. These two values are very close, so we simulated the spray velocity for the cases of d_1_ = 0.5 mm and d_1_ = 0.6 mm to make a comparison.

**Figure 3:**
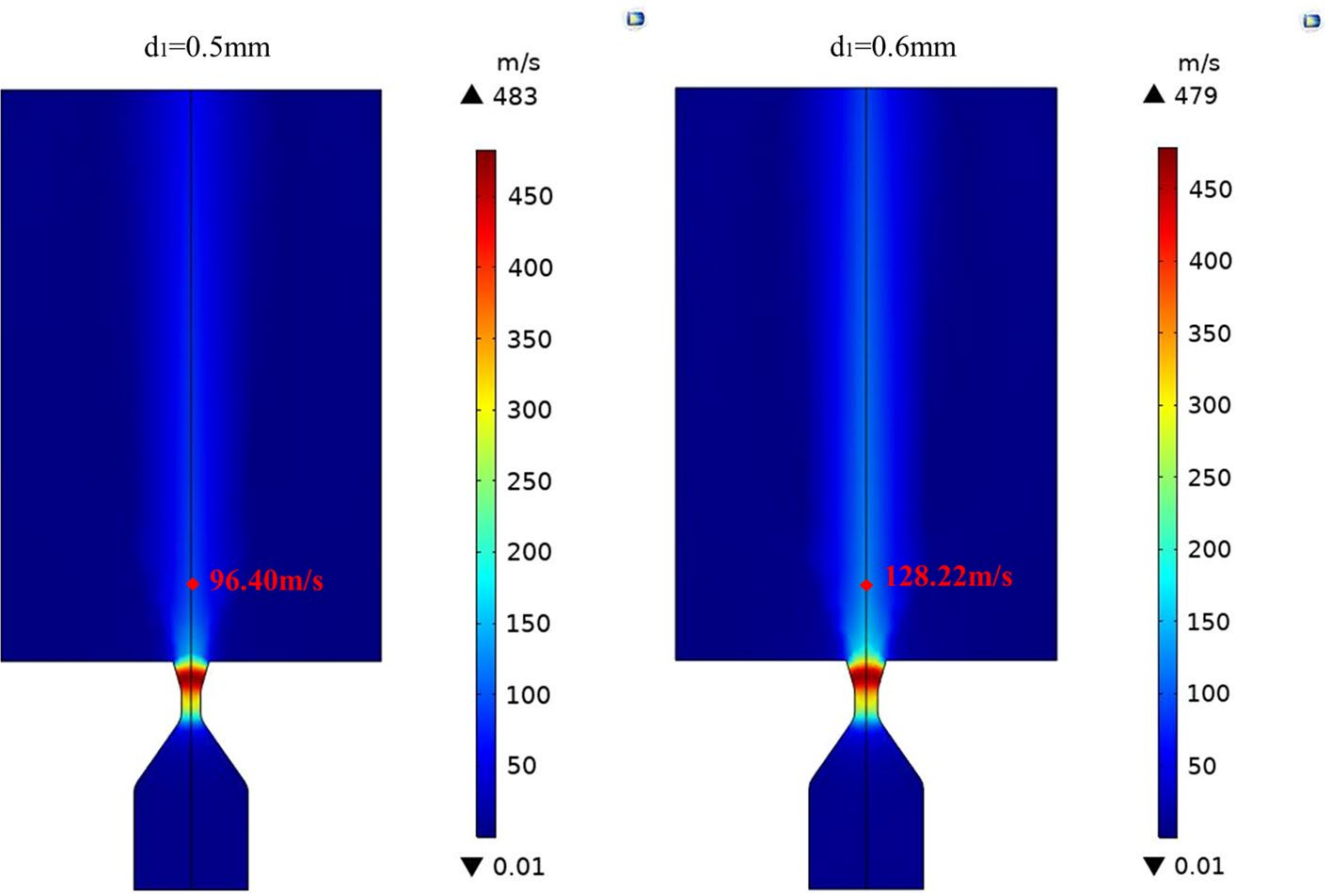
Simulation results of airflow velocity.

In the simulation model, the liquid channel is removed and the pressure of the airflow inlet is set to 2 bar. The simulation results are shown in Fig. 3. As can be seen from the figure, the maximum airflow velocity of these two Laval nozzles is 483 m/s and 479 m/s, respectively. The maximum velocities both appear at the position of the expansion section, and then the airflow velocity drops rapidly. Although the maximum airflow velocity when d_1_ = 0.6 mm is a little lower than that of d_1_ = 0.5 mm, the airflow velocity of d_1_ = 0.6 mm is not sufficiently reduced, and is still high in the middle region after the airflow ejected out the nozzle. At the measure point which respects the nostril, the airflow velocities are 96.40 m/s and 128.22 m/s, respectively. Since high spray velocity would cause nasal discomfort, d_1_ = 0.5 mm is selected as the preferred option.

### Spray angle

**Figure 4:**
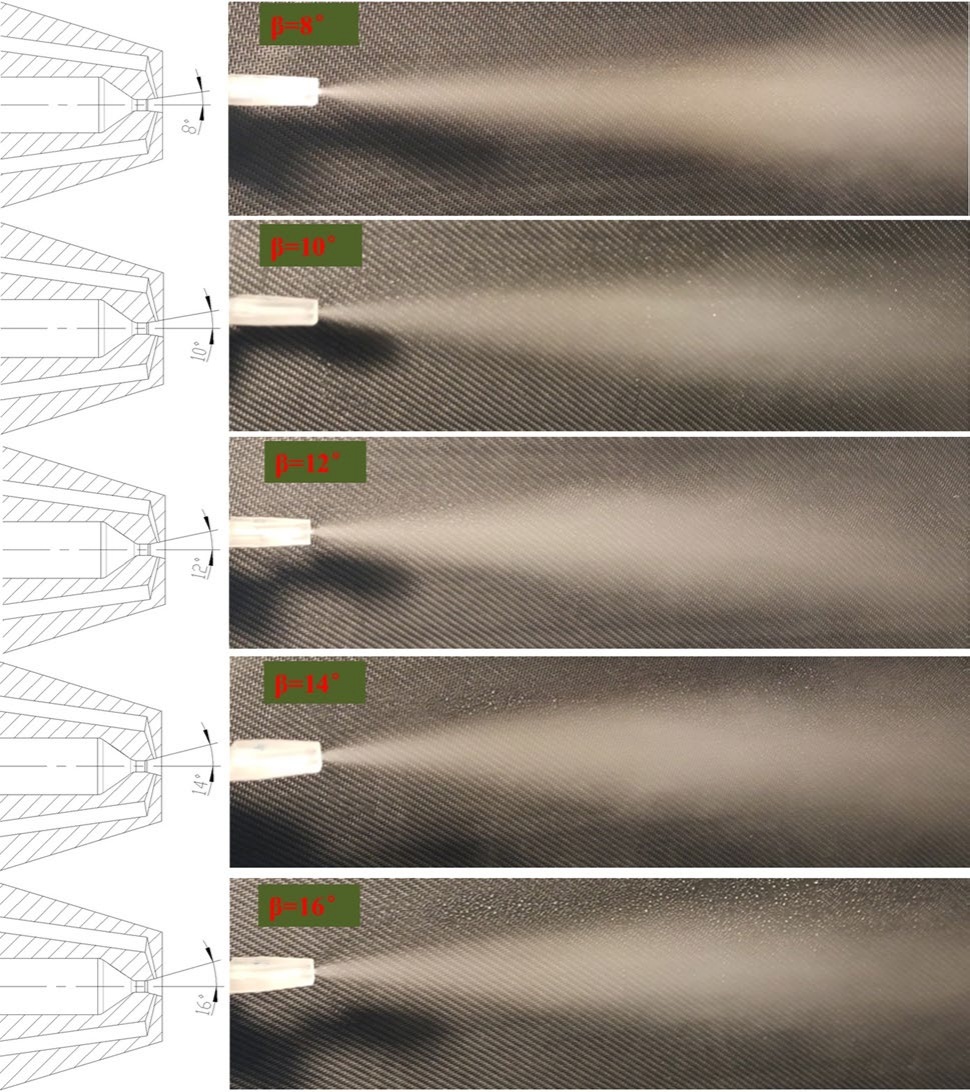
Spray angle of different β.

Herein, according to the univariate principle, it is assumed that the nozzle d_0_, α, d_1_, L_h_, d_2_ and γ are 3 mm, 35°, 0.5 mm, 0.5 mm, 0.1 mm and 75°, respectively. The air source pressure is 2 bar. The test results for Laval nozzles with different β are shown in Fig. 4.

As can be seen from the pictures, there is no significant change in the spray angle when the β is changed from 8° to 16°, the spray angle is all about 15°. Since the nozzle is close to the nostrils when vaccination, a spray angle of 15° is sufficient for the vaccine to enter the nostrils.

Based on the results above, the optimal Laval nozzle parameters are selected as follows: d_0_ = 3 mm, α = 35°, d_1_ = 0.5 mm, L_h_ = 0.5 mm, d_2_ = 0.1 mm, γ = 75°, β = 16°.

### Influence of air source pressure

**Figure 5:**
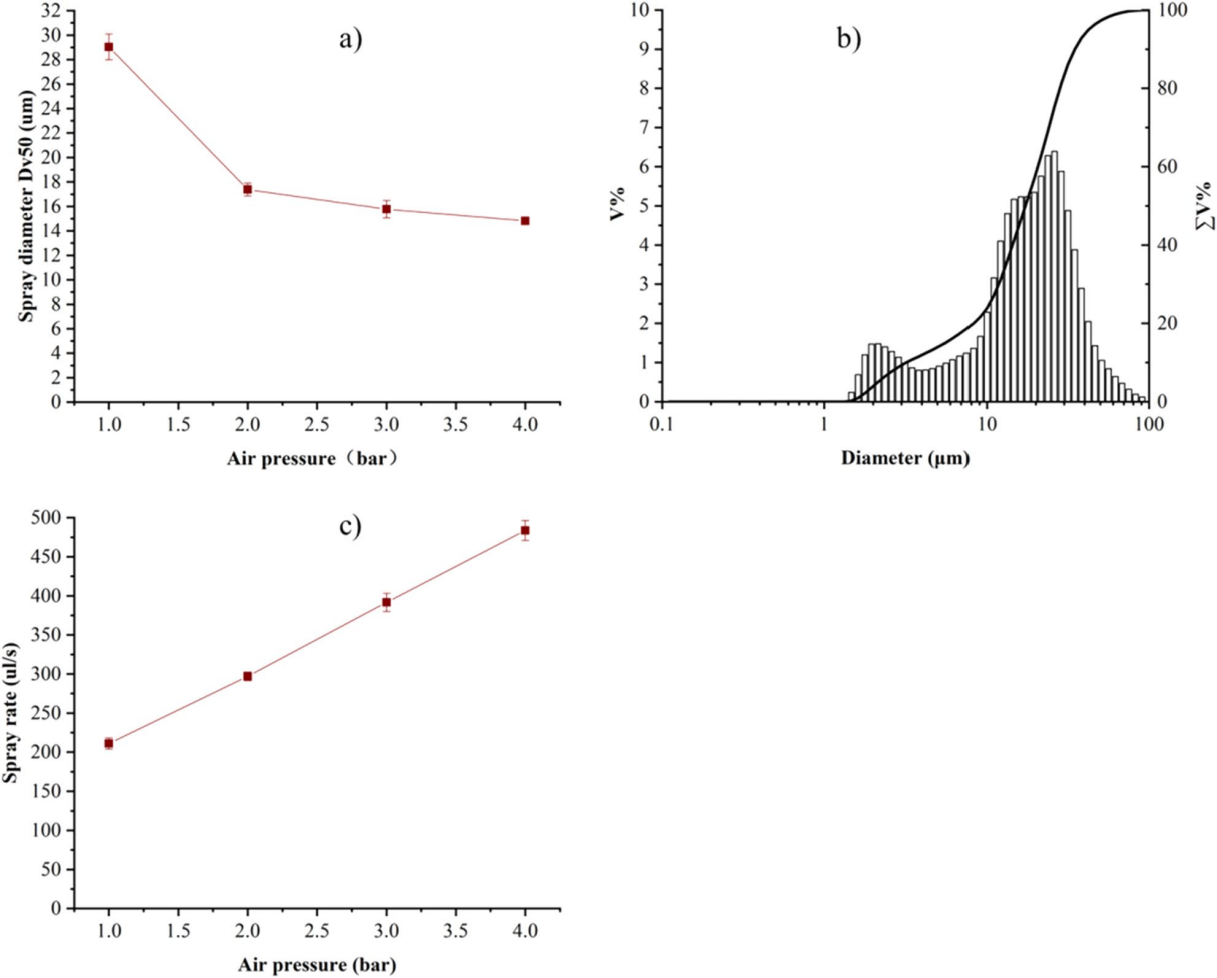
(**a**) Variation of spray particle diameter Dv50 at different air source pressure; (**b**) the spray particle diameter distribution at 2 bar air source pressure; (**c**) variation of spray rate at different air source pressure.

The air source pressure has a great influence on spray particle diameter and spray rate. To determine the influence, the spray results were tested at 1 bar, 2 bar, 3 bar and 4 bar air source pressure .The results of spray particle diameter Dv50are shown in Fig. 5a. It can be seen that theDv50 decreases gradually with the increase of the air source pressure, and the decreasing trend becomes smaller. Figure 5b illustrates the spray particle diameter distribution when the air source pressure is 2 bar. In the figure, Dv10 = 3.207 μm, Dv50 = 17.293 μm, Dv90 = 34.820 μm, 23.88% volume fractions of the particles are with particle diameter smaller than 10 μm. The effect of different air source pressures on the spray rate is shown in Fig. 5c. It can be seen that the spray rate increases gradually with the increase of the air source pressure, when the air source pressure is 2 bar, the spray rate is close to 300 μl/s.

### Vaccine activity test result

**Figure 6:**
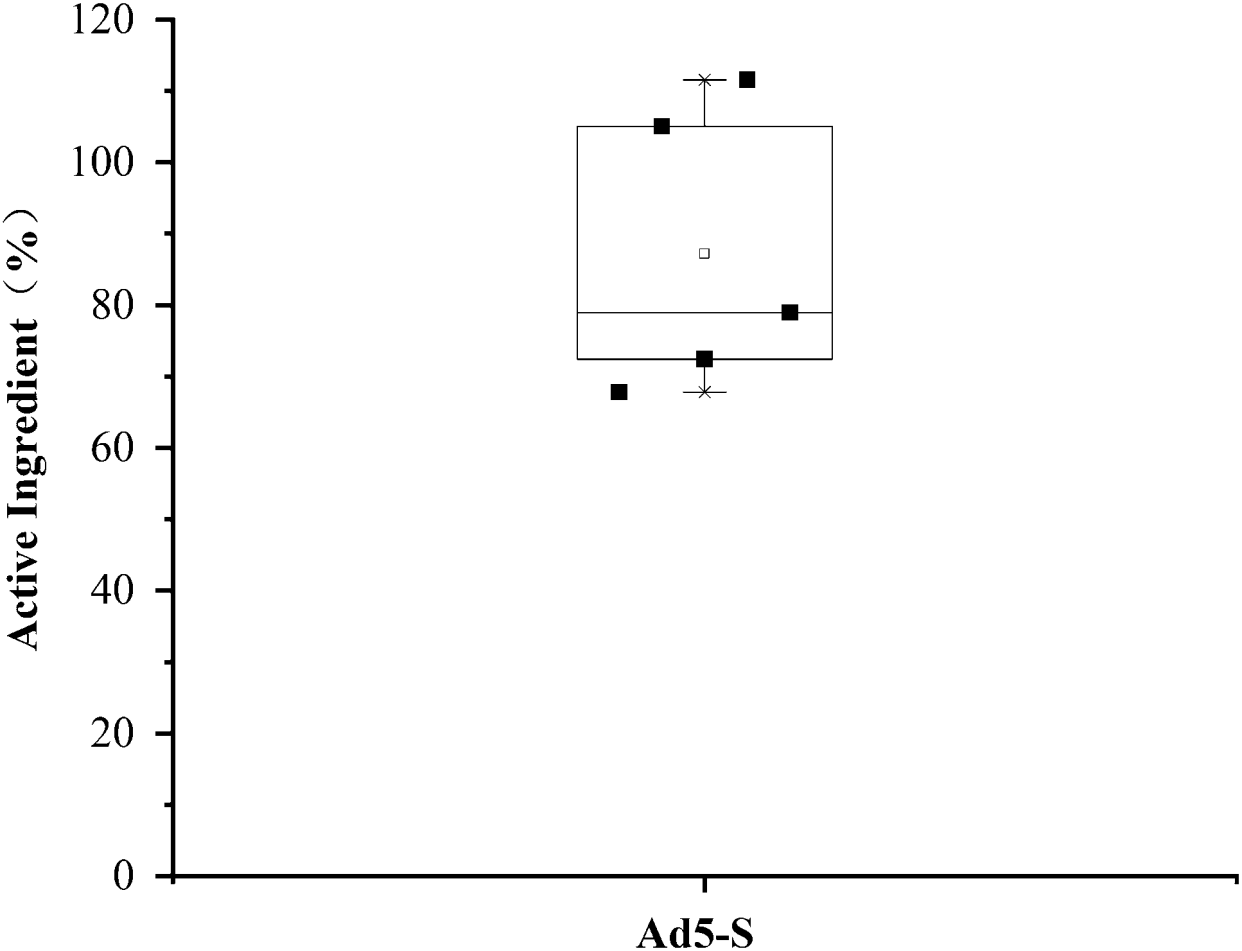
Ad5-S vaccine activity test result.

We compare the vaccine activity before and after passing through the device. Laval nozzle with optimal parameters was used for the tests. We find that the vaccine activity presented by IFU retained 87.2%, which indicating that the Active pharmaceutical ingredient (API) of the vaccine is well preserved after nebulization. The vaccine activity test result is shown in Fig. 6.

## Discussion

The surface roughness and accuracy of the 3D-printed nozzle should affect the spray performance. Since the nozzles in this article are printed by the same 3D-printer under the same parameters, so we assume that the influence of surface roughness and accuracy can be ignored. If the surface roughness and accuracy of the nozzle can be determined, the spray performance can be more assured.

Since we used vaccines with densities and viscosities very close to those of water (1.01 g/ml and 3.1 mPa s for vaccines and 1 g/ml and 2.98 mPa s for water, respectively), water was used to test particle size in this paper. For liquid reagents with different properties, their spray performance will be different. As an exploration we also measured the particle size of alcohol and Isopropyl alcohol, the results show that with the same condition, when the Dv50 of water is about 17 μm, that of alcohol and Isopropyl alcohol are about 10 μm and 12 μm, respectively. The relevant date is shown in the Supplementary Information. Generally speaking, a higher viscosity will case a larger spray particle size. When the air source pressure is 2 bar, the spray particle diameter Dv50 is about 17 μm, about 24% volume fractions of the particles are with particle diameter smaller than 10 μm, the airflow velocities at the nostril reaches 96.40 m/s, and the spray rate is already close to 300 μl/s. However, when the air source pressure reach to 4 bar, the spray particle diameter Dv50 is only reduced by 3 μm, while the spray rate comes to about 480 μl/s, causing more particles smaller than 10 μm and the airflow velocity at the nostrils also becomes higher. For the balance between the requirements of spray particle size and comfortable, 2 bar pressure is more suitable.

Although the airflow velocity of the optimized nozzle at the nostril is 96.40 m/s, which is still relatively fast, it doesn’t cause discomfort in the actual test because the spray time of each vaccination is less than 1 s, benefitted by the spray rate of 300 μl/s.

For different spray particle size requirements, it can be easily achieved by changing the air source pressure. To make the device more flexible, a small air pump integrated in the device can be used to instead of the nitrogen cylinder, an example of the prototype is shown in the Supplementary Information. Also, to avoid cross-contamination during vaccination, it is better to use a disposable shield when vaccination.

There are several commercial manual nasal spray devices such as BD Accuspray Nasal Spray System (BD Medical, Franklin Lakes, NJ, USA) and MAD Nasal Instranasal Mucosal Atomization Device (Teleflex, Morrisville, NC, USA). After testing, the Dv50 of spray particles of these two devices is about 60–70 μm. In comparison, the diameter of the spray device we designed is closer to 10 μm, which is better for delivery efficiency.

## Conclusion

In this paper, a nasal spray vaccination device based on Laval nozzle is designed, and the influence of different nozzle structures and different air source pressure on the spray particle size, spray rate and spray angle are analyzed. Through orthogonal experiments, it is demonstrated that the throat diameter d_1_ has the greatest influence on the spray particle diameter Dv50, and the optimal structural parameters of the Laval nozzle are: the diameter of the central tube d_0_ = 3 mm, the semi-cone angle of contraction section α = 35°, the throat diameter d_1_ = 0.5 mm, the throat length L_h_ = 0.5 mm, the reagent outlet diameter d_2_ = 0.1 mm, the reagent outlet angle γ = 75°, and the semi-cone angle of expansion section β = 16°. When the air source pressure is 2 bar, the Laval nozzle with this structural parameter has a spray angle of about 15°, the spray particle diameter Dv50 is about 17 μm, the volume fraction of the particles with diameter smaller than 10 μm is about 24%, and the spray rate is close to 300 μl/s. Ad5-S vaccine activity test results show that the IFU retained 87.2%, which indicating that the API of the vaccine is well preserved after nebulization.

Because of its ease of use, good nebulization performance and high vaccine activity rate, the device has wide application prospects in the prevention and protection of respiratory infectious diseases (“Supplementary Information”).

## Supplementary Information

Supplementary Information.

## Data Availability

The datasets generated during and/or analyzed during the current study are available from the corresponding author on reasonable request.
