# Supplementary material for: A nasal spray vaccination device based on Laval nozzle and its experimental test
Source: Sci Rep. 2023 Apr 17;13:6267. doi: 10.1038/s41598-023-33452-0 (PMC10109226; doi:10.1038/s41598-023-33452-0)
Supplement: Supplementary file 1 — Supplementary Information. [file 41598_2023_33452_MOESM1_ESM.docx]

With the same condition, when the Dv50 of water is about 17um, that of alcohol and Isopropyl alcohol are about 10um and 12um, respectively.


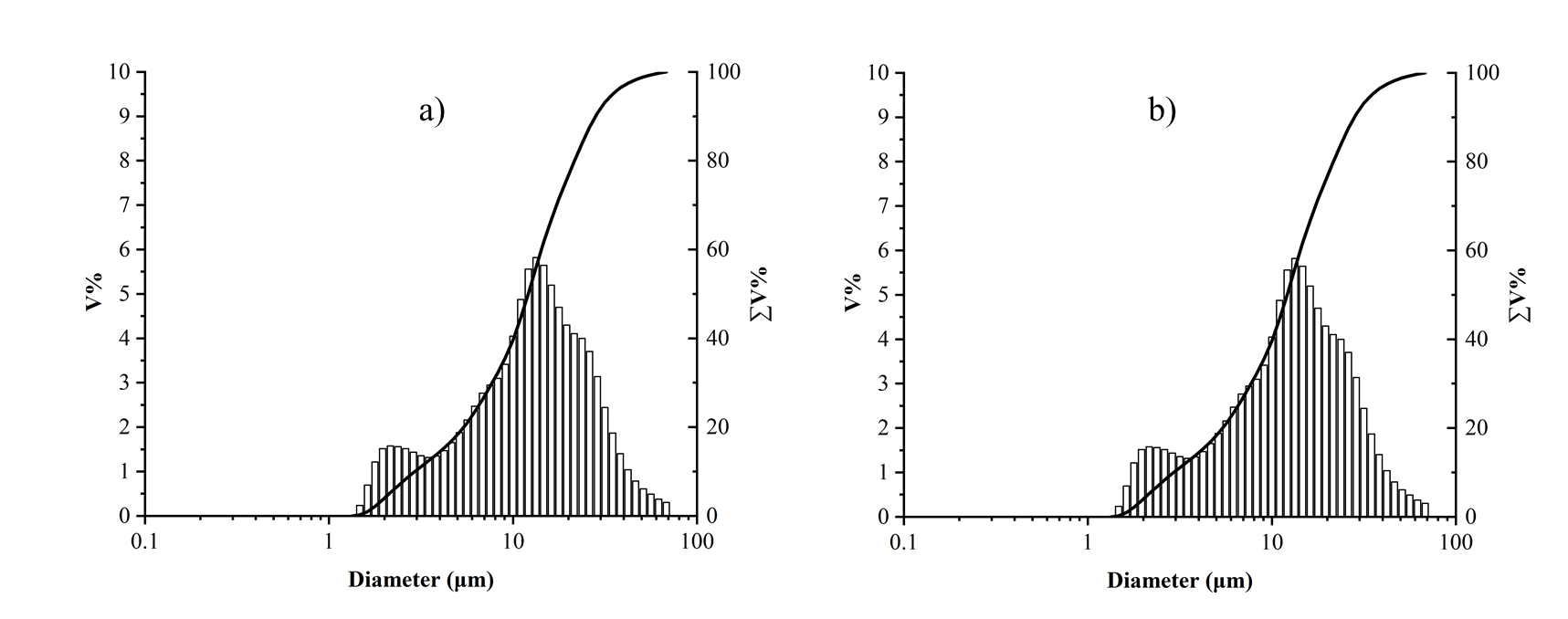


(a) the spray particle diameter distribution of alcohol; (b) the spray particle diameter distribution of Isopropyl alcohol


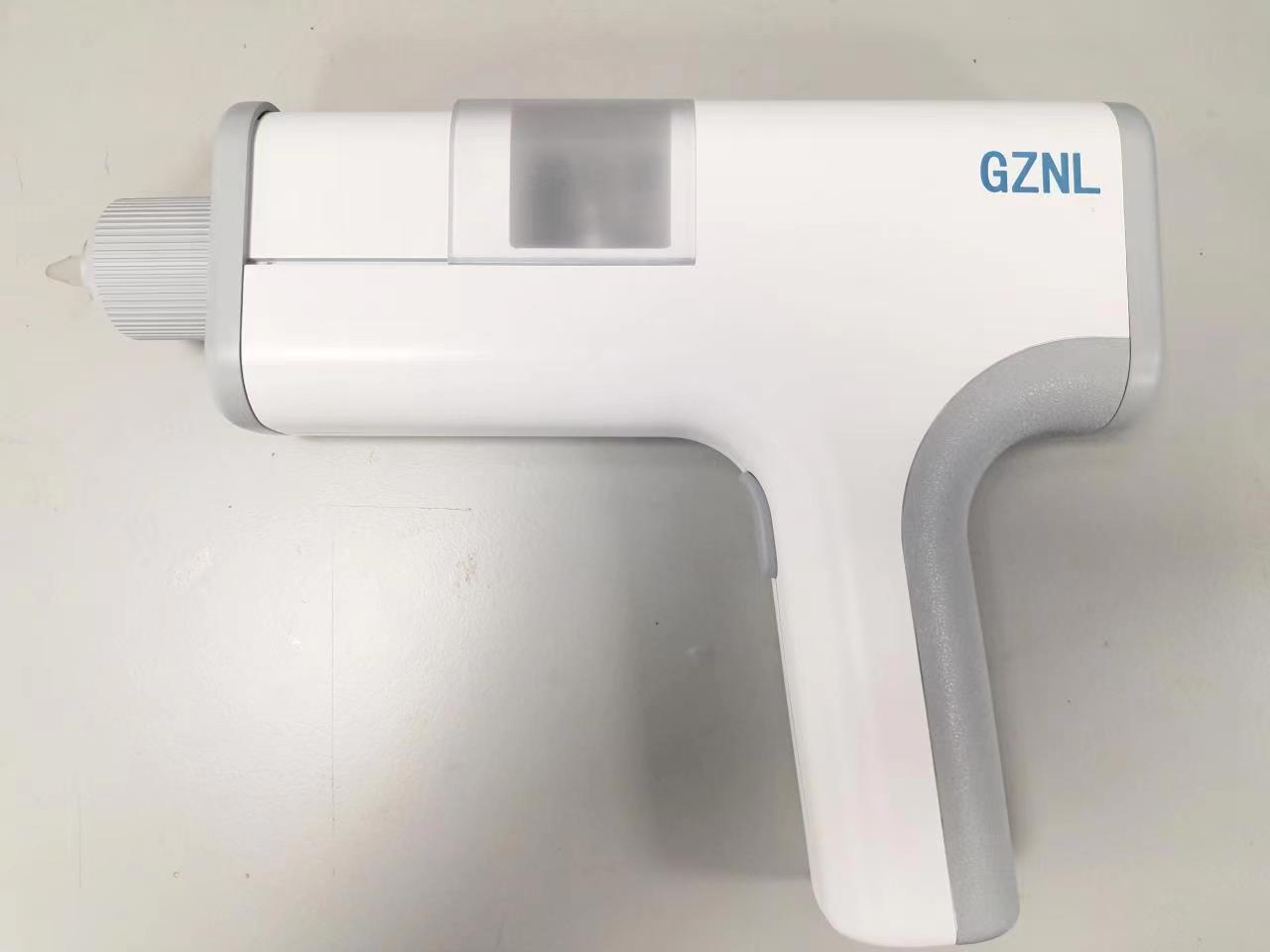


A nasal spray vaccination device with a small pump integrated in it
